# Mass and Charge Measurements on Heavy Ions

**DOI:** 10.5702/massspectrometry.S0074

**Published:** 2017-12-26

**Authors:** Toshiki Sugai

**Affiliations:** 1 Department of Chemistry, Faculty of Science, Toho University

**Keywords:** mass and charge simultaneous measurements, charge detection mass spectrometry, ion mobility spectrometry, ionization methods, large and heavy system with many charges

## Abstract

The relationship between mass and charge has been a crucial topic in mass spectrometry (MS) because the mass itself is typically evaluated based on the *m*/*z* ratio. Despite the fact that this measurement is indirect, a precise mass can be obtained from the *m*/*z* value with a high *m*/*z* resolution up to 10^5^ for samples in the low mass and low charge region under 10,000 Da and 20 *e*, respectively. However, the target of MS has recently been expanded to the very heavy region of Mega or Giga Da, which includes large particles and biocomplexes, with very large and widely distributed charge from kilo to Mega range. In this region, it is necessary to evaluate charge and mass simultaneously. Recent studies for simultaneous mass and charge observation and related phenomena are discussed in this review.

## INTRODUCTION

In the study of mass spectrometry (MS), almost all information is obtained through the ratio of *m*/*z*. It is typically diﬃcult to obtain mass and charge independently. For example, M^+^ and M_2_^2+^ are recognized as the same species in mass spectra.^1,2)^ However, in terms of ions with a smaller charge, it is possible to speculate each amount. Those multiply charged species (M_2_^2+^) appears as a series of peaks involving (M+2H_2_)^2+^ and M_3_^2+^ for example between those of singly charged ions. The intensities of each charge state show different dependences on conditions of ionization and sampling.

As a result, speculation is only possible when the resolution of MS is sufficient to resolve the *m*/*z* ratio of every ion and if its charge is relatively low. Recently ultrahigh mass species, such as biocomplexes, particles, cells, and viruses have been gradually incorporated into the scope of MS.^3–5)^ For those species, the charge and its distribution are large and the *m*/*z* resolution is not sufficiently high to permit perfect resolution so that their mass cannot be determined only by observing *m*/*z*. Some a combination of techniques such as ion mobility spectrometry (IMS) or the simultaneous observation of mass and charge so called charge detection mass spectrometry (CDMS) are indispensable. Here we report recent progress made in IMS combined MS (IMS/MS), tandem IMS, and CDMS, especially for particles and large molecules to obtain their actual mass and charge.

Mass and charge simultaneous observations have been performed since Millikans’ historical work.^6,7)^ Even in this very early stage of studies concerning elementary particles, a simultaneous measurement was crucial. His historical works can be viewed as a simultaneous measurement of charge, gravity, and mobility on a single particle. It is significant that the *m*/*z* of an electron was already determined before Millikans’ studies.^8)^ The fact shows that the determination of each of mass and charge is more difficult than that for *m*/*z*. However once the elementary charge was determined, the *m*/*z* of ions is almost equivalent to their mass itself with their speculated charge. This is one of the reasons why MS is widely accepted as a basic identification method. The method was developed in order to detect “differences” between samples and to resolve “complicated mixtures” with a high-resolution and a high-sensitivity. From this point of view the charge distribution must be reduced to unity or to a low a value as possible. The *m*/*z* resolution has been enhanced to as high as 10^5^ but a similar level has not been accomplished in terms of the charge as well as mass resolution itself.

The target of MS has now been extended to ultrahigh mass species, as mentioned above, for large biocomplexes and particles, where a large charge of around 100 *e* is on the molecule with a mass of around 1 MDa.^9,10)^ In this mass region, it is not sufficient to identify them with only based on *m*/*z* values. Other techniques such as IMS must also be employed.^9,10)^ Another approach is to determine the charge directly or indirectly. Recent progress on detection devices enables us to perform simultaneous measurements on molecules or particles in terms of *m*/*z* value and charge or kinetic energy.^1,4,5,11,12)^

For those measurements, ionization and detection methods are also important. It is difficult to desorb larger molecules and particles into a vacuum or gas and to detect them as their mass and size increase. Those species have strong adhesive forces arising from van der Waals interactions between wide flat contact areas, which prevent them from being desorbed. Their detection efficiency also is degraded because the electron multiplier,^13,14)^ the most common and effective detector, has diminished detection efficiency for low speed heavy ions. Not only detection efficiency but also manipulating the movement of those ions is a significant problem. Thermal energy and gravity are comparable to the kinetic energy of ions that are electronically accelerated when their *m*/*z* is high.

To treat larger species with MS, methods for the ionization of larger charges, enhanced detection methods, separation or identification methods have been in continuous development. Several of these achievements have been made: CDMS and IMS related measurements. Both methods utilize not only a sensitive electron multiplier but also low sensitive detection methods such as a charge detector and a condensation particle counter.^15)^ Despite their low efficiency, throughput, and sensitivity, those methods have many advantages, especially for larger species over electron multipliers that are used in conventional MS.

These current technical progresses and observed phenomena are reviewed here.

## EXPERIMENTAL TECHNIQUES

Experimental techniques have been developed to handle heavy ions. We summarize these techniques in terms of ionization, separation, and detection below.

### Ionization methods

#### Conventional methods

Many ionization methods have been developed for use in conjunction with MS so far. There are several problems associated with ionizing heavy and large molecules: large charge, desorption, soft ionization, and constant ionization efficiency. Laser desorption ionization (LDI) and matrix-assisted LDI (MALDI) are among the most suitable methods for ionizing samples with low volatility and with a very small amount such as a spot. Especially MALDI with the indirect ionization mechanism makes it possible to produce intact ions derived from large biomolecules.^16)^ However the charge is usually restricted to unity. It is very difficult to produce multiply charged ions by MALDI.^17)^ The detection efficiency for heavy molecules decreases as their mass increases when their charge is restricted to be low.^13)^ The results show that the ion detection efficiency is not unity, even in the relatively low *m*/*z* region under 10^4^. For very large biocomplexes or particles, MALDI may not be appropriate in terms of the amount of charge. Not only the charge, but also the ionization efficiency dependence on the matrix^2,18)^ and fine molecular structure^19,20)^ are also serious problems. There are many fields where the efficiency needs to be constant for all samples. To minimize the matrix effect, for example, a femtosecond laser has been utilized for elementary analyses.^18)^

Electrospray ionization (ESI) has also been shown to be a powerful method for ionizing especially biomolecules in very “soft” conditions.^21,22)^ Multiply charged ions are effectively produced by the method with highly sensitive detection even in high-mass region because of their reduced *m*/*z* with the high charge. Recently ultrahigh mass proteins,^9,10,23)^ nano or micrometer droplets of water or ethylene glycol,^24–27)^ polymers,^28,29)^ submicrometer polystyrene particles,^30,31)^ and even viruses^32–34)^ have also been produced by ESI and have been detected by MS and IMS with high charge up to 10^6^ *e*. Especially sonic spray a sort of spray technique with an electric field and a gas flow can also produce highly charged triethylene micrometer particles.^35)^

ESI thus has already realized the production of highly charged large and heavy molecules and particles. However charge and polarity are closely related to solution conditions such as pH and solvent.^2)^ It is necessary to control the conditions for some selected targets so as to generate sufficient charge. The large charge distribution accompanied by the large charge often makes it difficult to identify the mass of the samples only by *m*/*z* values. Handling of insoluble and trace amounts of samples on a tiny spot are also serious problems of ESI.^23)^

#### Laser induced acoustic desorption ionization

**Figure 1:**
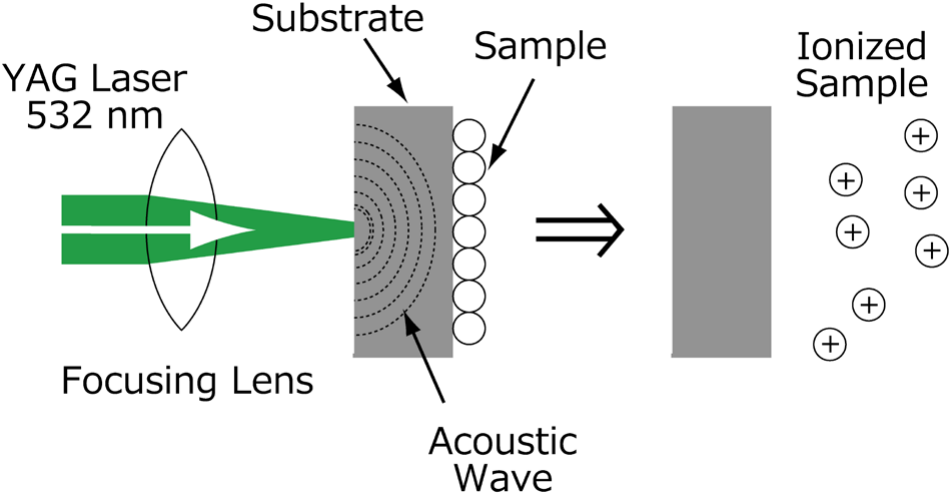
Fig. 1. Schematic view of laser induced acoustic ionization (LIAD).

Laser-induced acoustic desorption ionization (LIAD) is a method that attempts to solve the above mentioned problems.^36–43)^ Figure 1 shows a schematic view of LIAD. A fundamental or a second harmonic YAG laser beam with a relatively high power of 30 mJ/pulse is mildly focused on a sample substrate of sapphire plates separated by a layer of liquid mercury,^36)^ silicon wafers,^40–43)^ foils made of metals such as Fe, Al, and Ti.^37,38)^ The thickness of the plate is 500 μm for Si wafers and around 10 μm for metal foils. Samples such as cells, viruses, and large particles are deposited on the opposite side of the focus side of the sample plates and are then irradiated by the YAG laser. The power density at the point of focus is around 10^8^ to 10^9^  W/cm^2^. The laser pulse induces an acoustic pulse that desorbs and ionize the samples on the substrate. The ionization mechanism has not been well understood^36)^ but the efficiency is strongly dependent on the plate material.^38)^ Au and Si provide a high signal intensity but Fe does not. Some kinds of charge transfer or polarization between the sample and the plate should play crucial roles.^36,40–43)^

The polarity and the amount of charge of the produced ions are strongly dependent, not only on the sample plate, but also on the samples and the method used in their preparation. For large particles and heavy biomolecules, ions with a large positive charge are mainly produced.^36,43)^ The laser does not hit the sample directly so that the method is indirect and a very soft ionization method that can be applied for biosystems.^40)^

Thus indirect ionization, however, also results in a low ionization efficiency.^39,44)^ Sometimes LIAD is used mainly for desorption followed by post ionization such as electron impact, chemical ionization, and corona discharge. In terms of corona discharge, the charge is enhanced by one or two orders of magnitude.^40)^ The discharge is produced inside the trap just after the ion injection from LIAD by applying a high RF field of 3,000 V_pp_ for one second with the introduction of 50 mTorr of extra He. The discharge is monitored by observing the blue and white plasma light of the discharge and by measuring the charge of by-produced ions. The pressure is optimized in every measurement to achieve efficient ionization. With careful control of the discharge, the charge enhancement procedure is performed so as to not destroy the ions that are trapped. Those post ionization methods have always been the cause of the destruction of ions but the samples measured in this system are restricted to the large and heavy samples, such as cells which are sufficiently durable to hold their structures against the discharge.

The produced ions are heavy and their amount and distribution of charge are also large. To identify the sample, the determination of both of charge and *m*/*z* is indispensable, which is described in the sections of “Ion Trap” and “Direct Charge Detection.”

#### High-voltage assisted laser desorption ionization

To produce highly charged ions with a laser, high-voltage assisted laser desorption ionization (HALDI) is one of the candidate methods.^45–48)^ Multiple charged ions are produced effectively with HALDI^45)^ whereas single charged ions are produced in the case of LDI and MALDI.^16,17)^ In terms of polarity, with LDI and MALDI, both positive and negative ions are produced but the polarity is strongly dependent on the matrix.^2,18)^ HALDI can control the polarity of the product ions simply by the voltage.^45,46)^ Another advantage of HALDI is that no matrix is used, which leads to signals without contaminating ions from the matrix.^45)^

**Figure 2:**
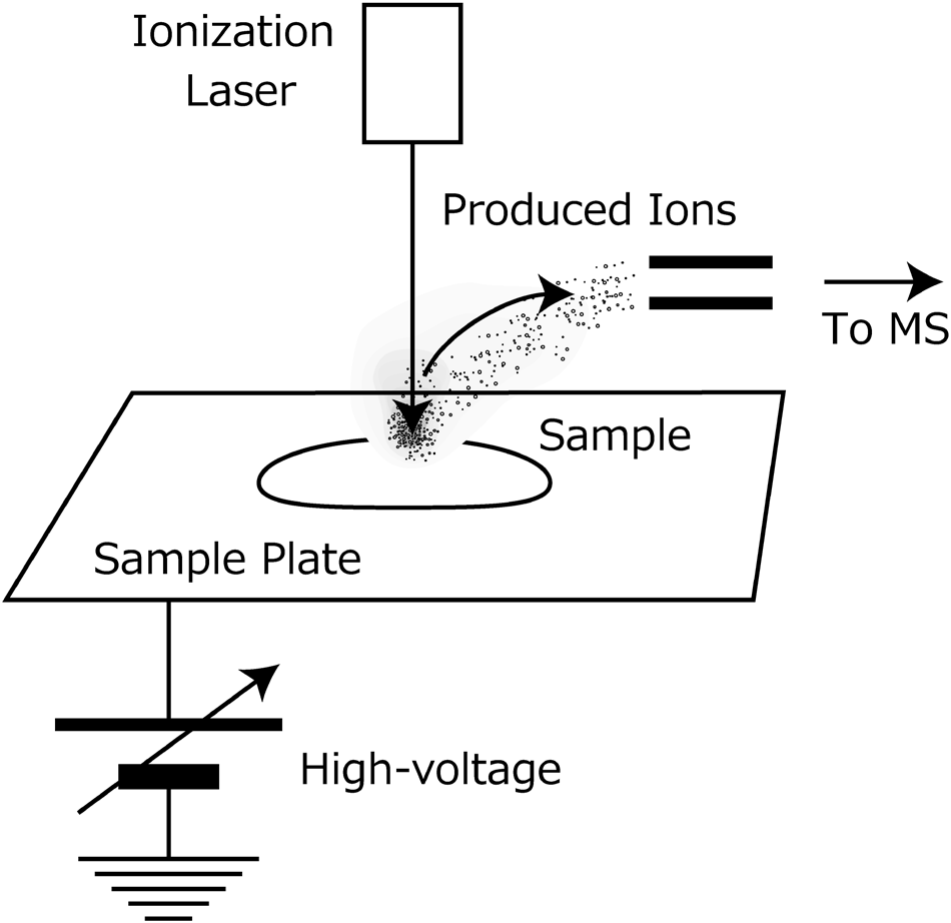
Fig. 2. Schematic view of high-voltage assisted laser desorption ionization.

Figure 2 shows a schematic view of HALDI, which consists of an ionization laser, a sample plate, and a high-voltage power supply. A fundamental beam of a YAG laser is used to desorb and ionize sample solutions on the sample plate made of a conductive material such as Au, Al, Ag, and stainless steel, a semiconductive material of Si, and nonconductive one of polytetrafluoroethylene (PTFE) where a high-voltage (HV) of several kV of both polarities is applied.

Large biomolecules such as cytochrome *c*, ubiqutin, and myoglobin are ionized to have large charge over +20 *e* without any matrix, which are similar to ions produced by ESI, from solutions of the sample in methanol/water (50 : 50, v/v) with 0.1% formic acid.^45,49)^ Ions are produced only when both the high-voltage and the laser are applied. The signal intensity heavily depends on the laser power used, the HV, the pH of the solution, and the materials of the sample plate. The high-voltage applied to the sample plate produces a high-field at the laser spot that contributes to the ionization of the sample. In terms of the plate material, no ion is produced with nonconductive PTFE but is produced in the case of semiconductive Si. The authors proposed that some roughness of the plate contributes to the ionization processes. The pH dependence is similar to that of ESI: the average charge number increases from +8 of the methanol/water solution without formic acid to around +17 of that with 0.1% formic acid. The method can be considered to be a hybrid method of LDI and ESI with the advantages of both.^45)^

### Separation

#### Ion trap

**Figure 3:**
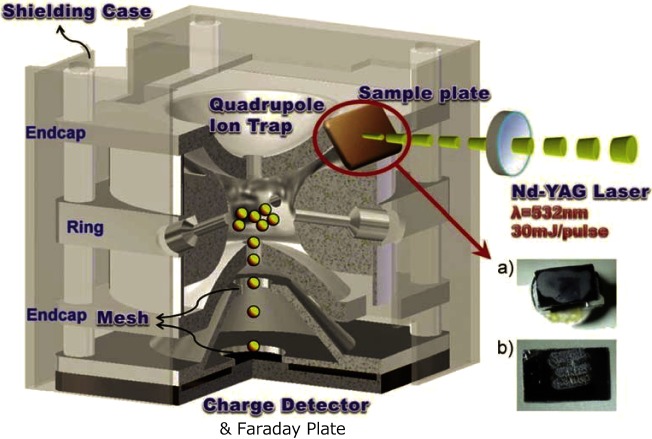
Fig. 3. Schematic view of a direct charge monitoring mass spectrometer with a quadrupole ion trap, mesh, charge detector & Faraday plate, and a laser-induce acoustic desorption ionization source.^4)^ Reprinted with permission from Royal Society of Chemistry. Copyright (2013) Royal Society of Chemistry.

An ion trap, which was originally developed to cool down high-energy particles,^50–53)^ is now used in MS.^2,4,51,54,55)^ Various kinds of ion traps have been developed to hold, separate, and break ions.^2,55,56)^ One of the most common ones is a quadrupole ion trap. Figure 3 shows the structure of the trap for heavy ions^4)^ with the LIAD ion source described in the previous section, and a charge detector with a Faraday plate that is described in the “Direct Charge Detection” section. The system consists of a quadrupole ion trap, LIAD, the detector with the Faraday plate, and a mesh. The mesh is made of stainless steel with more than a 90% transmission and is placed between the Faraday disc and the exit hole of the ion trap to shield the RF field of the ion trap. For usual MS, a high-frequency (10^5^ to 10^6^ Hz) high-voltage (100 to 300 V) (RF) is applied to the center ring-like electrode. To apply the quadrupolar potential, the electrodes have hyperbolic surfaces with minimum distances of *r*_0_ and 
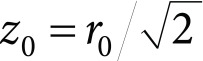
 in terms of the radius and the axis. The shapes of the electrodes provide an inhomogeneous field distribution in the trap where the field near each electrode is strong whereas that further away is weak. At the center of the trap, there is no field of RF.

When the produced electric field changes too quickly for the ions to follow, the ions near the electrode gradually move to the place apart from the electrode to avoid the high field. Finally the ions are trapped at the center of the trap where the field is lowest.^51,52)^ The movement of the ions in the trap strongly depends on the *m*/*z* of ions and the frequency and voltage of the RF. To hold heavy ions with larger *m*/*z* against gravity, a lower frequency and a higher voltage of around 100 Hz and 1 kV are crucial.^4,40–43,57)^ However, when a too low frequency and a too high voltage are applied, ions with low *m*/*z* can follow the field and the amplitude of the movement in the trap is large enough to be ejected from the trap.^51–53)^ This frequency and the voltage dependence is represented mathematically as the stability condition, which is derived from the Mathieu equation: 
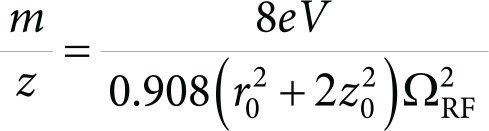
(1) where *V* and Ω_RF_ is the amplitude and the angular frequency of the RF. When we apply 1,000 V and 200 Hz to the trap with the size of *r*_0_=10 mm to Eq. (1), the lowest *m*/*z* of ions to be held is estimated to be 2.7×10^9^.

Usually *m*/*z* scanning is performed by increasing *V* with a fixed Ω_RF_ . The scanning range in this case is limited to three orders of magnitude because of limitations associated with the RF power supply. In contrast, a much wider range of more than ten orders of magnitude is realized by scanning Ω_RF_.^4)^ The frequency and the voltage dependence is utilized as *m*/*z* separation. For usual ions with low *m*/*z* the scanning is performed with increasing RF voltage to eject ions with *m*/*z* values from lower to higher. In this *m*/*z* region, the frequency tends to be high around 1 MHz where the RF power supply works in the resonant condition with the fixed frequency. It is easy to change the RF voltage but not the frequency. For ions with higher *m*/*z*, on the other hand, a frequency of around 100 Hz is sufficiently low to permit audio electronic devices with broadband characteristic working at nonresonant conditions to be used.^4,40–43)^ The *m*/*z* selection to eject from lower to higher *m*/*z* can be performed by scanning the frequency from higher to lower. Actually, the *m*/*z* separation of heavy ions such as cells, viruses, and large particles is performed with the system in Fig. 3. With the scanning from 350 to 20 Hz with a voltage of several kV, various polystyrene particles, cells, and viruses with *m*/*z* from 10^7^ to 10^11^ can be readily separated.^4,44)^

**Figure 4:**
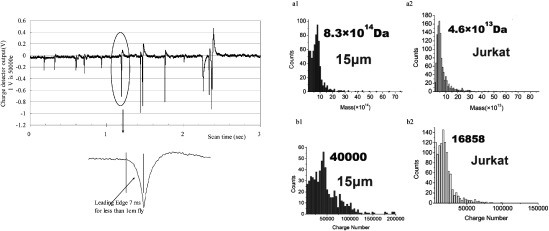
Fig. 4. (Left) Mass spectrum of Jurkat cancer cells.^41)^ Each peak corresponds to the charged cell with an average mass of 4.6×10^13^ Da. The horizontal axis shows a scanning time of a trap RF frequencies from higher to lower, which corresponds to the *m*/*z* around 10^9^ from lower to higher. The vertical axis shows the voltage of a charge sensitive amplifier described in Fig. 7. The expansion shows a pulse shape of a peak. (Right) Constructed mass and charge histograms of polystyrene particles and Jurkat cancer cell.^41)^ Reprinted with permission from American Chemical Society. Copyright (2008) American Chemical Society.

Figure 4 (left) shows the observed mass spectrum of Jurkat cancer cells^41)^ ionized by LIAD with post ionization of the corona discharge, as described in the “Laser Induced Acoustic Desorption Ionization” section. Each peak corresponds to the charged cell.^41)^ The horizontal axis shows the scanning time of the RF frequencies from higher to lower corresponding to *m*/*z* values from lower to higher. The vertical axis shows the voltage of the charge sensitive amplifier described in the “Direct Charge Detection” section. The expansion shows a pulse shape.^41)^ Figure 4 (right) shows the constructed mass and charge histograms for polystyrene particles and Jurkat cancer cells^41)^ from information regarding each ion on *m*/*z* and charge. The procedure to determine mass and charge of each sample is described in the “Direct Charge Detection” section. The obtained average mass and charge are 8.3×10^14^ Da and +40,000 *e* for the polystyrene particles with a diameter of 15 μm and 4.6×10^13^ Da and +16,858 *e* for Jurkat, respectively. The mass of the particle of 8.3×10^14^ Da is in good agreement with the expected one of 1.1×10^15^ Da.

**Figure 5:**
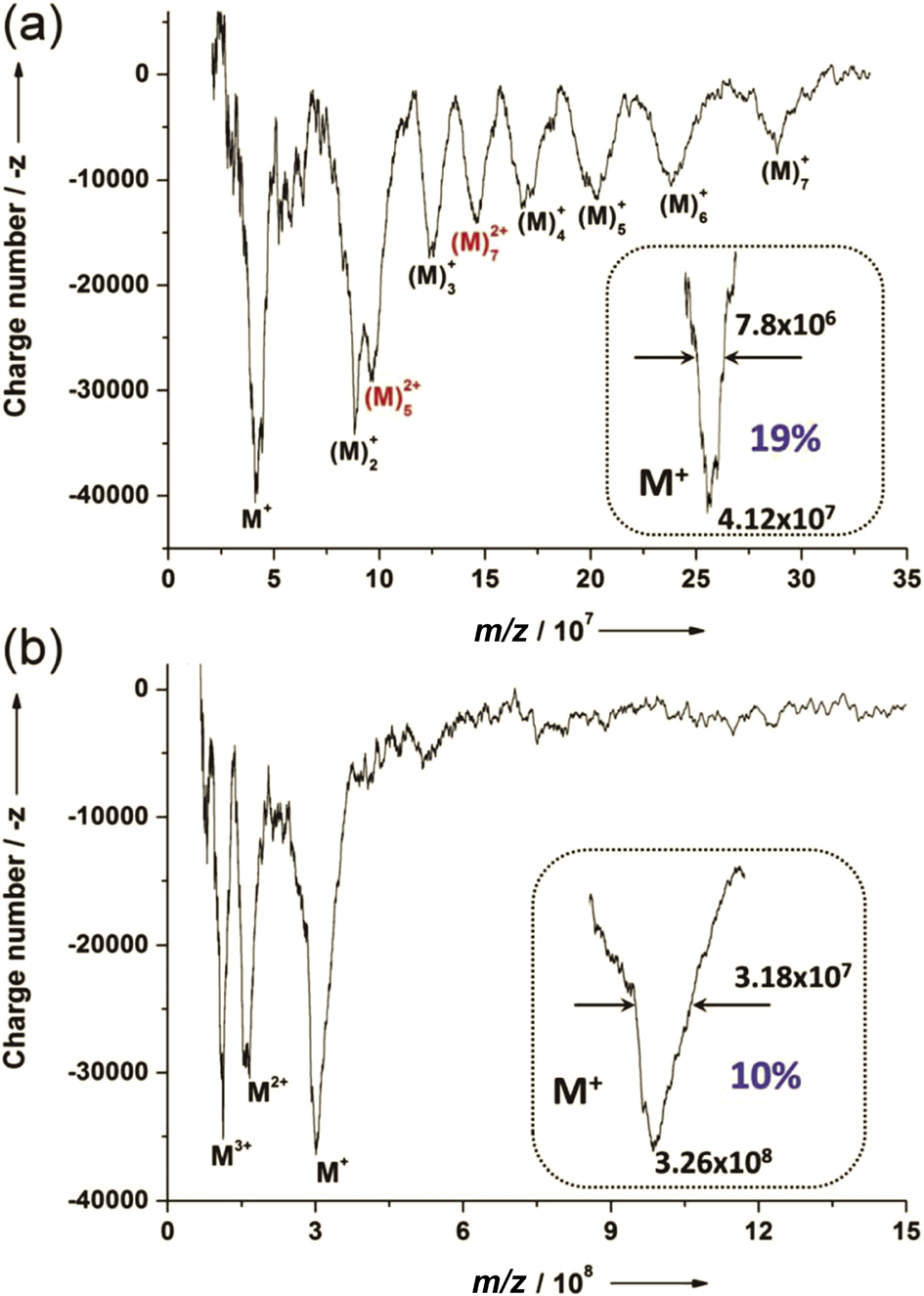
Fig. 5. Mass spectra of standard polystyrene particles with diameters of (a) 50 and (b) 100 nm, respectively.^4)^ The horizontal axis represents *m*/*z* but most of ions are singly charged so that the axis is identical to mass. The vertical axis represents the charge number. The peak area of each peak for singly charged ions corresponds to the number of ions. The expansions show peak widths of the monomers with a single charge. The resolutions are estimated to be around 5 and 10, respectively.^4)^ Reprinted with permission from Royal Society of Chemistry. Copyright (2013) Royal Society of Chemistry.

The *m*/*z* and mass resolution is evaluated with C_60_ and standard polystyrene particles with accurate, predefined diameters from 50 nm to 3 μm. In the case of C_60_ with the RF frequency of 200 kHz in 20 mTorr He, a mass resolution of (*m*/*z*)/Δ(*m*/*z*)≈500 is achieved.^40)^ Approximately 15,000 singly charged C_60_ ions are trapped here detected with the Faraday plate. The *m*/*z* and Δ*m*/*z* are evaluated by the conversion from frequency to mass with Eq. (1). Figure 5 shows the mass spectra of the standard polystyrene particles with the diameters of 50 and 100 nm.^4)^ There are many species with multiple charges and clusters. The horizontal and vertical axes represent *m*/*z* and detected charge, respectively. For the singly charged ions *m*/*z* is equivalent to *m*. Apart from Fig. 4 with very few ions of much larger cells and particles, Fig. 5 shows measurements that seem to be performed with many ions, each peak area corresponds to the number of detected ions. The expansions show the peak widths of monomer ions with a single charge. The peak tops appear at 4.12×10^7^ and 3.26×10^8^ Da, which are in good agreement with the expected values of 4.14×10^7^ and 3.31×10^8^ Da. The FWHM widths are 19% and 10% of the corresponding peak top mass for the 50 and 100 nm particles so that the FWHM mass resolutions are estimated to be around 5 and 10. Because the mass distribution of the standard particles are estimated to be around 3% with the narrow diameter distribution of 1%, the observed wide mass distribution must be due to the resolution of this system. Thus, the obtained much low resolution compared to that of C_60_ may not arise from noise of the detection system described in the “Direct Charge Detection” section, because even for much heavier ions with a large charge, the *m*/*z* and mass resolution is restricted to be 4 where the noise level is not dependent of the charge of ions.^4,40,41)^ In these kinds of trap measurements, the pressure in the trap can be a serious problem. Here 20 to 100 mTorr helium buffer gas is used for the heavy ions to dump the initial kinetic energy to hold ions at the center of the trap and to enhance the charge with the corona discharge. This relatively high-pressure may cause a reduced resolution for heavy ions but for C_60_, it is irrelevant.

Those results show that heavy ions such as cells, viruses, and large particles can be analyzed by MS with a relatively high *m*/*z* resolution. However *m*/*z* observations are not sufficient to determine mass. The mass of the ions can be only determined by observing *m*/*z* and *z* simultaneously and precisely. In addition, the *m*/*z* analysis with high-sensitivity for the heavy ions requires ions with a large charge to detect them as a single molecule and also some special ionization methods such as LIAD with corona discharge. The large charge leads to a large charge distribution and low resolution of the charge measurement, which results in difficulties in determining the charge and the mass precisely. The precise determination the charge of a single molecule is still challenging subject to solve.^31,58,59)^

#### Ion mobility spectrometry

The resolution of MS reaches the level of 10^5^, which is sufficient to resolve large biocomplexes up to 1 MDa with tens of *e*. However the large charge distribution coming from the large charge as described above results in mass spectra that are overlapped and complicated in terms of determining the actual mass. To solve the problem of MS, IMS is now becoming a key technology as a combined system of IMS/MS where IMS works as a structural filter or separator for MS.^2,53,55)^

IMS can separate the structures of ions through intermolecular interaction or collision between ions and buffer gas.^2,53,55,60,61)^ The selection is performed based on mobilities, which closely related to the cross section between ions and buffer gas or their size. The mobility is tightly correlated to *m*/*z* values, but is not identical to it.^2,55,60)^ IMS/MS provides us with so called “two-dimensional” information of *m*/*z* and mobility on each ion from which we can deduce the precise mass of ions. For example, M^+^ and M_2_^2+^ have the same *m*/*z* but different mobilities because they have different structures.^2,53,55)^ In practice, the collision, which is essential to IMS, causes the diffusion or loss of ions and signal intensities. In addition, the collision requires a much higher pressure of buffer gas than that of MS, which leads to difficulties in interfacing IMS to MS. Despite of those difficulties, there are many IMS systems that can be connected to MS. Here we introduce two types: differential mobility analyzer (DMA) and traveling wave IMS (TWIMS).

**Figure 6:**
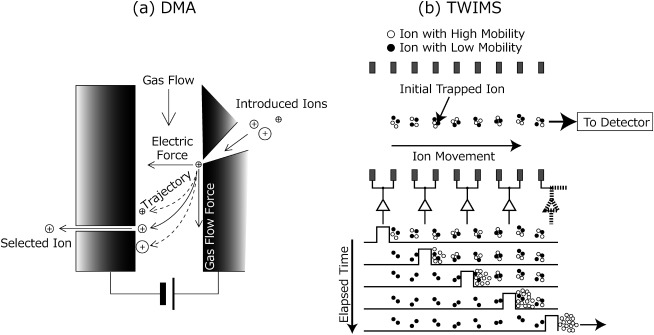
Fig. 6. Schematic view of IMS. (a) Differential mobility analyzer (DMA). (b) Traveling-wave ion mobility spectrometer. The dotted lines show repeated and extended structures.

Figure 6 shows structures of (a) DMA and (b) TWIMS. Both are commercialized and have many applications.^2,53,55)^ DMA working at ambient pressure has been used as a filter for aerosol and particles for many years,^61)^ which selects and passes some ions with a certain mobility. It utilizes two electrodes with an electric field between them and the presence of a gas flow, and an inlet and an outlet of ions. The introduced ions feel the electric force of the field and the force of the air flow. The resultant force and the ion mobility dictate the trajectory and the selection of ions inside DMA. Too large or too small ions cannot track the right trajectory to pass through DMA (Fig. 6a). Because DMA functions at ambient pressure basically, the order of measurements are always DMA first and MS second to follow the gas flow due to the difference between their working pressures.

TWIMS, on the other hand, was developed by Waters Co., Ltd.^62)^ to be integrated into MS so that the commercialized system has TWIMS between multiple MS systems. To connect MS systems easily, TWIMS functions at a very low pressure around several hundreds Pa compared to DMA. Figure 6b shows a schematic diagram and the basic concept of the system. The system is equipped with a stacked ring ion guide (SRIG) where a RF (around MHz and several hundreds V) is applied to co-axial ring electrodes to trap ions. Each adjacent electrode of SRIG has an opposite polarity of RF so that intense and high-frequency electric fields are applied between the nearest electrodes. The frequency is so high that the ions cannot follow the polarity change of the RF moving gradually from the inner rim to the center of the SRIG according to the field strength. The ions then are trapped at the center of the each electrode since the trap potential is cylindrically symmetric with sinusoidal distortion along the center axis.^62)^ The trap mechanism is similar to that for a quadrupole ion trap. The trapped ion can be easily transported by applying bias voltages along the axis in SRIG. To achieve a higher resolution and high sensitivity, the length of the trap should be long so that the SRIG structure is repeated to extend the length of the trap, which are represented as dotted triangle in Fig. 6.

The procedure of TWIMS is shown in Fig. 6b, where a waveform called a travelling wave (TW) is applied to the SRIG electrodes for ion mobility with the superimposed RF for the ion trap. TW is continuous moving waves with a field of tens of V/cm and a speed of hundreds of m/s, which drives the trapped ions out of the SRIG to be detected by MS. The ions with higher mobility represented by open circles in the figure are effectively transferred to MS whereas those with smaller ones represented by filled circles are left in the SRIG. Thus, the mobility is measured by the ejection efficiency in TWIMS. This procedure is crucial for TWIMS where whole ions are utilized for the mobility measurements in contrast to DMA which can only pass selected ions. High sensitivity with a reasonable resolution can be realized by tuning the voltage and the speed of the TW.

TWIMS has achieved high sensitivity with SRIG by applying not only a DC bias but also well-designed RF and TW waveforms. Those waveforms drive the ions with complicated trajectories and the high fields of the waveforms heat up and distort the ions.^62,63)^ These effects make it difficult to convert the observed ejection efficiencies or the time profile of TWIMS to the actual mobilities. For example, the linear relationship between the mobilities obtained from the TWIMS profile and the mobilities obtained by other measurement systems is fairly good for rigid and spherical molecules whereas it does not hold for asymmetric or soft molecules.^64)^

By utilizing both systems, it is possible to deduce the mass and charge of heavy biocomplexes and particles with many charges.^9,10,24,25,35)^ It is very difficult to distinguish heavy ions with many charges from a complicated one dimensional *m*/*z* mass spectrum. However the overlapped peaks in the spectrum can be separated in the two dimensional mass-mobility spectrum. The mass, charge, and also structure of the heavy ions with up to 1 MDa and around 100 *e* are determined precisely.^9,10)^

Those commercialized systems contribute to the rapid development of heavy biocomplexes with their structural and functional analyses. There is an essential problem associated with such studies: the structure must be stable and predictable. Those molecules can easily change their structures due to environmental changes such as the nature of the solution and the vacuum, and to intra-charge repulsion with many charges.^2,53,55,61)^ When the structure is unstable and unpredictable, it is not possible to correlate series of mobility peaks to those of charge numbers. The charge is speculated from the mobility obtained in IMS measurements.

To avoid the difficulties associated with multiple charges, charge reduction has been utilized, especially in IMS studies.^24,25)^ Charge reduction is performed by means of a “neutralizer” with radioactive materials such as ^241^Am where small positive and negative ions produced by the radiation become attached to sample ions with multiple charges through the attractive Coulomb force between them. Finally the number of the charge of the sample ions is reduced to 0 or ±1 as described to be “neutralizer” literally. The neutralizer is located between two IMS spectrometers in tandem IMS systems. The first spectrometer selects ions with some mobility, which can have various sizes and charges. To determine the size, the charge is reduced to unity by the neutralizer followed by the second mobility spectrometer. In the second IMS, the size is determined from the fixed unit charge.

In this case the problems associated with multiple charges are resolved but a new problem associated with the small charge appears. The sensitivity of the electron multiplier or other detectors for the ions with small charge is too low to permit their use in usual MS. So condensation-particle-counters (CPC) are used for the detection. To be detected with light scattering in CPC, charged particles are enlarged by nucleation with an alcohol such as *n*-butanol on the particles in supersaturated atmosphere.^65,66)^

### Charge detection

#### Direct charge detection

Charge itself has not been typically observed in MS. The accelerated high-energy ions are introduced to an electron multiplier which produces millions of secondary electrons in the device to be detected as a pulse current. We can then count the numbers of ions through the pulse current but usually not the charge. Recent progress in electronic devices makes it possible to directly observe the charge of a single ion.^4,44)^ This technique is particularly useful, not only for determining mass from *m*/*z* but also for detecting heavy ions themselves where the sensitivity of the electron multiplier such as SEM and MCP is dramatically degraded because of small velocities even for highly accelerated ions.^13,14)^ Significant progress has been recently made on techniques for charge detection for being developed as charge detection mass spectrometry (CDMS), which was originally developed in 1960.^4,5,59,67)^

Charge sensitive amplifier (CSA) itself has a long history. Its main application is to resolve energy of radiation such as gamma rays with a semiconductive detector where the energy of the radiation is proportional to the number of electron-hole pairs or the induced charge.^14)^ There are several commercial CSA such as A250 (AMPTEK) and SD-37 (Hamamatsu) with sensitivity as low as several hundreds *e*/mV as the ratio between the input charge and the output voltage. The sensitivity is not sufficient to permit ordinary MS to detect ions with one or two *e* but is applicable to the detection of multiply charged heavy ions.^4,40–43)^

**Figure 7:**
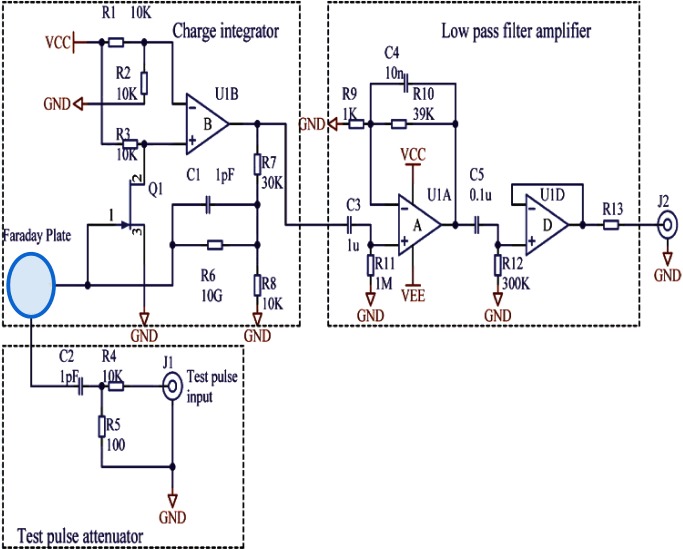
Fig. 7. Schematic diagram of a charge sensitive amplifier with a charge integrator, a low pass filter, a test circuit, and a Faraday plate.^41)^ The Faraday plate is represented as a white blue ellipse on the left side. The charge integrator detects and accumulate image charge at the Faraday plate induced by ions to be detected. The low pass filter amplifies the signal and reduces high-frequency noise. The test pulse attenuator provides a function of the calibration of the charge sensitivity of the amplifier. Reprinted with permission from American Chemical Society. Copyright (2008) American Chemical Society.

Figure 7 shows a schematic diagram of CSA connected to the detector for heavy ions shown in Fig. 3.^41)^ As described in the “Ion Trap” section, the ions with the selected *m*/*z* by tuning the RF frequency are ejected from the trap and introduced to hit the detector which consists of a disk with a diameter of 1 cm as the Faraday plate. The amplifier detects the charge through the plate and outputs a voltage pulse with the height that is proportional to the charge. The pulse shape is shown in Fig. 4.^41)^ The mass and charge are simultaneously determined through *m*/*z* and *z* obtained from the peak position of the ejected timing and from the peak height, respectively.^41)^

CSA consists of a charge integrator, a low pass filter amplifier, a test pulse attenuator, and the Faraday plate represented as a white blue ellipse (Fig. 7), which corresponds to the plate in Fig. 3.^41)^ The charge integrator detects the charge and accumulates the image charge at the Faraday plate induced by ions to be detected. The low pass filter amplifies the signal and reduces high-frequency noise. The test pulse attenuator functions to calibrate the charge sensitivity of CSA. The sensitivity is determined by the transmittance of Q1 and capacitance of the gate, C1, C2, and the Faraday plate, which are strongly dependent on parts to parts fluctuation and the working temperature of the CSA. To calibrate the sensitivity for a precise ion charge evaluation, a fixed voltage pulse is applied to the test pulse input. The test pulse attenuator is designed so as to inject the charge of 63,000 *e* to the Faraday plate with a test pulse of 1 V. The test pulse is attenuated by 1/101 with R4 and R5 resistor dividers and the charge is injected through C2 of 1 pF. When we apply a test pulse of 1 V, a voltage difference of 10 mV is applied across C2 where the charge of *Q=C V=*1 pF×10 mV=10 fC=63,000 *e* is accumulated. With the circuit and the calibration, the charge conversion of 1 mV/50 *e* and the noise of 10 mV (rms), corresponding to about 500 *e* rms, are obtained.^41)^ However it is very difficult to determine the exact sensitivity, even with this test circuit, due to the fluctuation.

**Figure 8:**
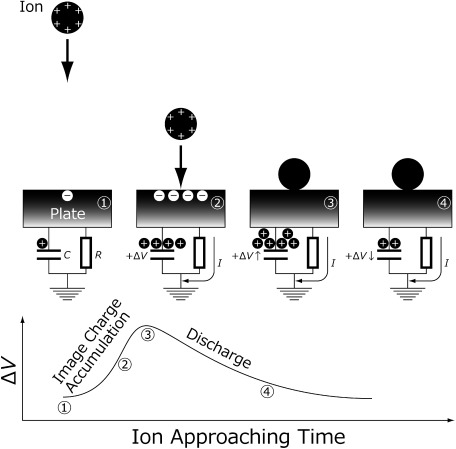
Fig. 8. Sequence of image charge accumulation and discharge while an ion is approaching to hit a plate to which a capacitor (C) and a registor (R) are connected from ground. Those C and R are the parts of the charge sensitive amplifier to detect the charge of ions shown in Fig. 7. (1) When an ion is approaching a metallic plate, an image charge is induced. The induced image charge compensates the field by the charge of the ion and it appears on the surface of the plate near to the ion. The counter charge of the image charge is collected on the capacitor. (2) As the ion approaches to the plate, the charge on the capacitor increases as well as the image charge. (3) The voltage on the capacitor increases until the ion hits the plate to neutralize the charge on the ion and the image charge. (4) After the collision, the charge and the voltage decrease gradually because of the discharge through the resistor. The ground voltage is not constant but is controlled by a feedback of the amplifier (see text).

The charge of an ion including its image charge is accumulated on the Faraday plate while the ion approaches the plate from the trap. The sequence of the charge accumulation and discharge is shown in Fig. 8. While a multiply charged positive ion approaches the conducting Faraday plate, the image charge (negative in the figure) to compensate the field induced by the charge of the ion (positive in the figure) appears on the surface of the plate near the ion. A capacitor (C) and a registor (R) are connected to the plate from the ground. Those are the key parts of the integrator of the amplifier (Fig. 7). The capacitance of the capacitor shown in Fig. 8 is actually distributed to C1, C2 and a gate (terminal 1) of Q1 in the integrator and the test circuits. The register R corresponds to R6. Because the capacitance of those parts is much larger than that of the plate, the counter charge of the image charge is collected on the parts represented as capacitor C in Fig. 8. As the ion approaches the plate, the charge on C increases as well as the image charge. The voltage on C increases until the ion hits the plate to neutralize the charge on the ion and the image charge. After the collision, the charge and the voltage on C decrease gradually because of the discharge through the resistor of R.

The voltage on the capacitor is sensed and amplified by Q1 of an N-ch junction field effect transistor (Nch-JFET) and U1B of a high-impedance low noise operational amplifier in the integrator (Fig. 7) followed by the low pass filter amplifier for further amplification and noise reduction. The amplification is performed “inversely” so that the actual outputs for positive ions are negative-going peaks with a period of around 10 ms (Fig. 4). The ground level of C and R is not always 0 V but actually is controlled by the feedback of the operational amplifier of U1B to keep “virtual short” between “+” and “−” terminals of U1B, which results in the actual amplification. Figure 8, however, shows the basic circuit operation mechanism.

CSA has many characteristics such as charge sensitivity, a discharge time constant, and a noise level. Charge sensitivity is one the most important characteristics of charge detection, which is determined mainly by the Q1 and C1 values for the integrator. To enhance the sensitivity, the total capacitance C should be as low as possible, since the signal voltage is defined as Δ*V*=*q*/*C* where *q* is the accumulated charge. For the detection of heavy ions from the trap, however, the capacitance *C* should not be too small because of the accumulation time constant τ determined by the product of the capacitance by resistance *R* of R6 as τ=*C*×*R*.^41)^ The accumulation time of the image charge is around ms due to the large plate size and slow ion velocity coming from the trap.^41)^ Thus the integrator must wait until the accumulation of the charge is complete, otherwise the accumulated charge would be lost. The long accumulation of 10 ms is achieved using large resistor of 10 GΩ of R6. With these customization for the heavy ions, the sensitivity of 50 *e*/mV and the noise level of 500 *e* are achieved.^41)^ The output voltage is basically proportional to the detected charge but the proportional factor or sensitivity always strongly depends on the devices on each amplifier and on the working temperature of the CSA. The calibration is also difficult because the production of the precise amount of charge is not so easy. Consequently the accuracy of the detected charge is not so high.

The method achieves high-sensitivity for single particle mass spectrometry but the charge sensitivity and accuracy are not so high. Further development will be necessary.

#### Indirect charge detection

Direct charge detection is a powerful method. However indirect charge detection for measuring “image charge” also has a long history. The method has been widely used in trap type mass spectrometry such as Fourier transform ion cyclotron resonance mass spectrometry (FTICR) and Orbitrap™ from Thermo Co., Ltd.^68,69)^ Both of the trap type MS methods have achieved high-resolutions of up to 10^5^ on *m*/*z*, which is very hard to be achieved by other methods. To achieve such a high resolution, Fourier transformation of ion movement in the trap is crucial. With a specially designed magnetic or electronic trap, the trapped high-energy ions continue long lasting periodical movements dictated by their *m*/*z*. The ultrahigh vacuum and the image charge detector realize the tracking of the movements.^68,69)^ From the Fourier transformation of the detected movements, whole ion species with different *m*/*z* values are identified as an intensity of each frequency component. This advantage contributes the throughput: all ions with different *m*/*z* can be analyzed from one track of the movements with Fourier transformation. To detect all species with good *S*/*N*, the frequency and the frequency distribution of the movements should be high, around MHz, requiring the use of a high-frequency broadband CSA to detect image charge because the resolution and sensitivity increase with increasing number of cycles of the periodical movements.^68,69)^ Based on those features, this indirect charge detection has been developed to enhance the detection sensitivity and the resolution of charge evaluation for a single heavy ion with multiple charges up to viruses as indirect CDMS recently.^5,59,70–72)^

**Figure 9:**
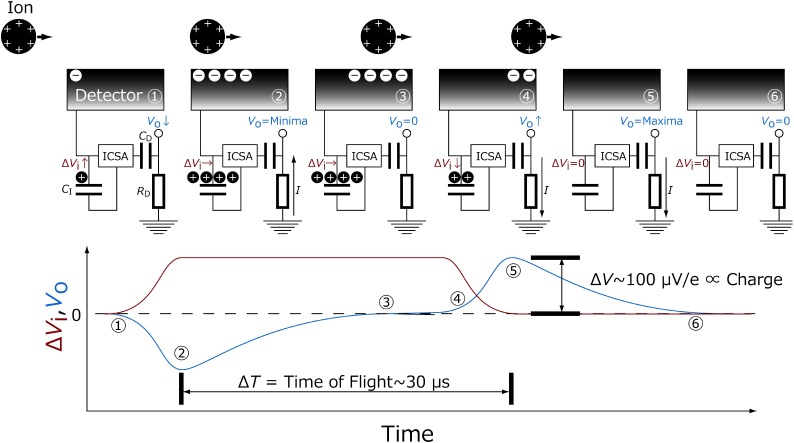
Fig. 9. Sequence of image charge detection while an ion passes through to a tube like detector (the bottom half is shown) where a capacitor (C_I_), an inverted charge sensitive amplifier (ICSA) are connected. The output of ICSA is connected to a sort of capacitor(C_D_)–registor(R_D_) differentiator. The upper of the figure shows the ion motion while the bottom shows the detector signal of Δ*V*_i_ (the voltage difference across C_I_) and the final output signal of *V*_o_ corresponding to the processes described in the upper. (1) The process starts with the ion entrance. (2) The image charge is accumulated and Δ*V*_i_ and *V*_o_ reaches maxima and minima, respectively. The sensitivity of ICSA is around 10–1,000 *e*/mV. (3) The magnitude of the image charge as well as Δ*V*_i_ is constant until the ion is inside the detector tube. The output of *V*_o_, on the other hand, decreases by the current through the registor of R_D_ to *V*_o_=0. (4) When the ion begins to leave the detector, the image charge and Δ*V*_i_ decrease resulting in *V*_o_ increase until (5) the ion finish to leave with *V*_i_ as 0 and *V*_o_ as maxima. (6) Finally the both voltages go to zero (Δ*V*_i_=*V*_o_=0) with the discharge. The amplitude and the peak to peak time difference are proportional to the charge and the time of flight of the ion, respectively. The ground level of C_I_ is not constant but is controlled by feedback of the amplifier (see text).

Figure 9 shows a sequence of the indirect charge detection.^28,29,58,73)^ A tube like detector (the half of bottom is shown here) to which a capacitor (C_I_) and an inverted charge sensitive amplifier (ICSA) are connected. The ICSA is similar to the circuit of Fig. 7, but the output of ICSA is connected to a sort of “differentiating circuit” to detect the precise timing of the ion motion.^28,29)^ The differentiator has many patterns of implementation but here is shown a simplified capacitor(C_D_)–registor(R_D_) differentiator. The inversion is mainly caused by Nch-JFET in Fig. 7 as the first stage of the charge amplifier. The results of the indirect CDMS presented here and in the following sections are obtained by an A250 (Amptek) with Nch-JFET, which is different from the circuit in Fig. 7. There are many circuit designs and implementations not to use the “inversion way.” However all the results are consistent with the inversion way so that ICSA is adopted throughout in this review.

The upper of the figure shows the ion motion while the bottom shows the input voltage of Δ*V*_i_ as the voltage difference across C_I_ and the final output signal of *V*_o_ corresponding to the processes described in the upper. The ion initially approaches the detector invoking an image charge (1). When the ion enters the tube detector perfectly, the image charge is accumulated and Δ*V*_i_ reaches maxima (2). The connected ICSA amplifies the detector signal inversely with a sensitivity of around 10–1,000 *e*/mV. The amount of the image charge as well as Δ*V*_i_ is constant until the ion is inside the detector tube (3). The output voltage of *V*_o_, on the other hand, decreases by the current through the registor R_D_ to *V*_o_=0. When the ion begins to leave the detector (4), the image charge and Δ*V*_i_ decrease resulting in an increase in *V*_o_ until the ion finishes to leave with Δ*V*_i_ as 0 and *V*_o_ as the maxima (5). Finally both voltages go to zero (Δ*V*_i_=*V*_o_=0) with the discharge (6). Here again, the ground level of C_I_ is not always 0 V but is controlled by the feedback. The amplitude and the peak to peak time difference are proportional to the charge and the time of flight (TOF) of the ion, respectively. The observed amplitude with the amplifier sensitivity of −24 *e*/mV is around 80 mV for the ion of polyethylene oxides with the charge of +1,900 *e*. The TOF observed for the same ion is around 30 μs with the velocity of 1,400 m/s.^28)^ The charge is proportional to *z* so that the charge is deduced directly from the amplitude, providing there is no noise.^28)^ On the other hand, TOF depends not only on the velocity gain of the ions by the electrostatic acceleration but also on the initial velocity of the ions (*v*_I_) induced by gas flow at an ESI source for example. Since the ions are so heavy, the effect of the initial velocity is large. For example, we get 830 eV as kinetic energy in the case of *m*=1 MDa and *v*_I_=400 m/s. Finally *m*/*z* is obtained from TOF as 
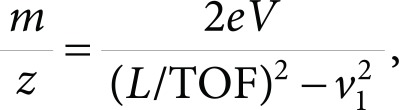
(2) where *V* is the acceleration voltage, *L* is the length of the detector tube.^28,74)^

Indirect CDMS measurement can be performed with this single-pass measurement.^28)^ Mass and charge information is deduced from the obtained *z* and *m*/*z*. However the charge information especially has large uncertainty because of the noise of the amplitude similar to the direct CDMS. To reduce the noise and the uncertainty of the charge information, averaging is the most effective technique. That can be performed by using many electrodes up to 22^74)^ or placing the detector in an ion trap.^5,58)^

**Figure 10:**
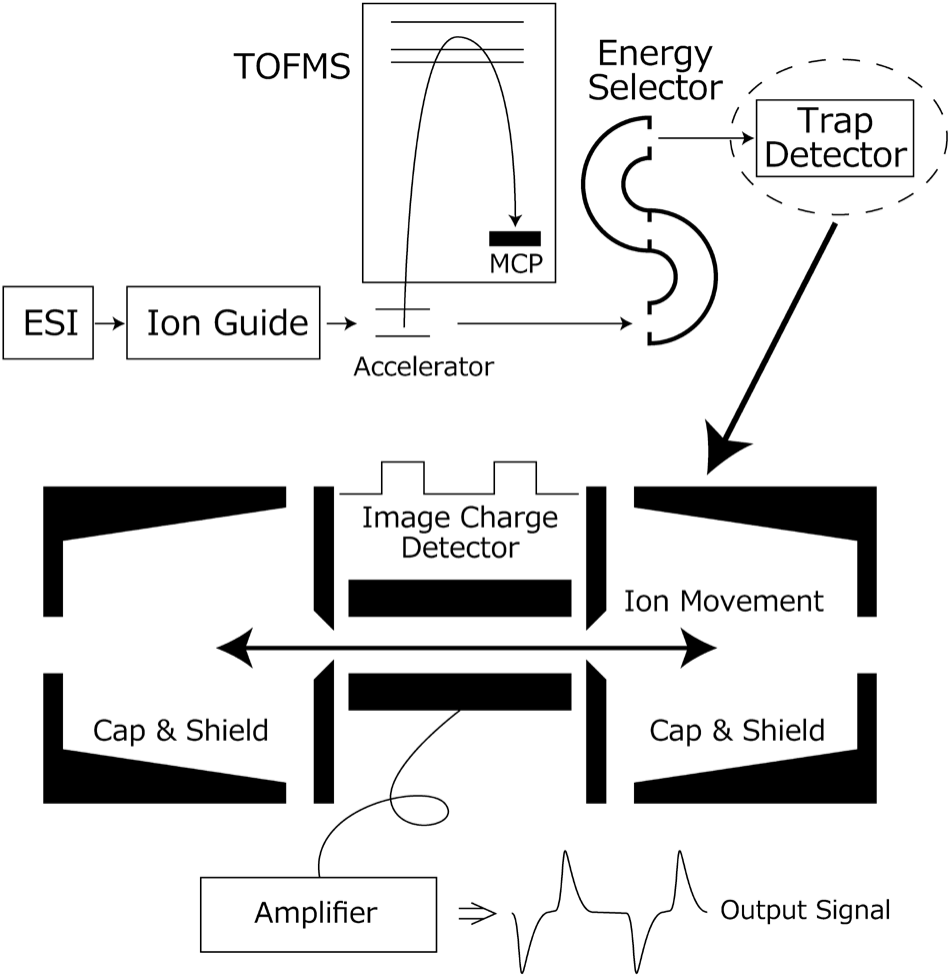
Fig. 10. Schematic view of a charge detection mass spectrometer.

Figure 10 shows a schematic view of the multi-pass CDMS with the trap detector. The system consists of an ESI ion source, an ion guide, a TOF, an energy selector, and a trap detector.^75)^ Ions produced with the ESI source are introduced to the energy selector through the ion guide and the acceleration region of the TOF mass spectrometer for temporal evaluation. Energy selection is performed with a dual hemispherical deflection analyzer (HDA) because the *m*/*z* measurement significantly depends on the kinetic energy of the ions. The HDA consists of two concentric hemispherical electrodes with different potentials having radii of 19.05 and 31.75 mm for the inner and outer hemispheres, respectively. Three apertures with diameters of 2.54 mm are equipped in the entrance, middle, and exit parts of HDA. To achieve high energy selectivity, the reduction of angular deviation of the ion trajectory, and the deceleration and acceleration of ions across HDA, and the ion focusing are employed. With these techniques, the energy resolution of 0.45% with a spread of 99.75–100.20 eV is achieved.^75)^ After the selection of ions with the kinetic energy of 100 eV/charge, the ions enter the trap detector through the hole shown in the expansion of the figure.^5)^ The potentials of the endcaps of the trap are periodically manipulated to hold and eject the ions with a cycle time from tens of ms to several seconds. The trapped ions oscillate in a field produced by shields and caps. In the oscillation, the ions enter and leave the center image charge detector with a tube-like structure, as shown in Fig. 9. With this motion the potential of the detector goes up and down through the charge accumulation and discharge processes as shown in Figs. 9 and 10.^73)^ Thus obtained detector signal is amplified (Fig. 9). To reduce noise, the Nch-JFET (Fig. 7) is cooled down cryogenically with liquid nitrogen and whole amplifier is located near the detector in the vacuum chamber. The temperature in a measurement is estimated to be 125 K.^71)^ The output signals are averaged to reduce the noise up to for several seconds or during several ten thousands of oscillations.

**Figure 11:**
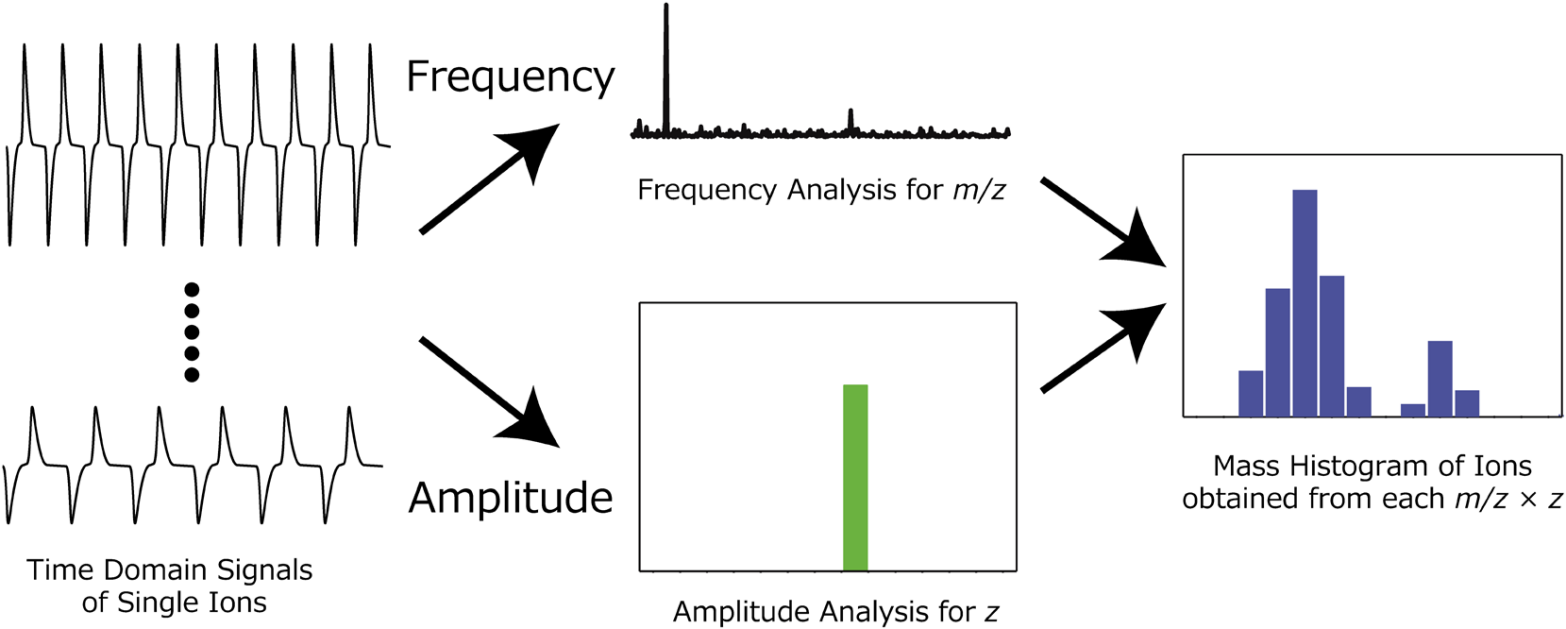
Fig. 11. Data processing from time domain signals to a mass histogram. The time domain signals of each measured ion are analyzed in terms of frequencies and amplitudes. The *m*/*z* is obtained from the frequency while the charge of *z* is obtained from the amplitude. The both data are combined to determine mass of *m* by multiplying *m*/*z* by *z*. Finally the mass histogram is constructed through the analyses of many ions.

The process from the thus obtained oscillations as time domain signals to a mass histogram is shown in Fig. 11. The time domain signals of each measured ion are analyzed in terms of the frequency and the amplitude. The *m*/*z* is obtained from the frequency while the charge of *z* is obtained from the amplitude. The data for both single ions are combined to determine the mass of *m* by multiplying *m*/*z* by *z*. Finally the mass histogram is constructed through the analyses on many ions. The *m*/*z* of ions is actually obtained from the fundamental frequency *f* of the oscillation and the selected energy *E* through the following equation^73)^: 
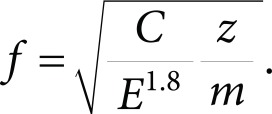
(3)

The dependence of *f* on *E* in Eq. (3) is not proportional to 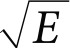, which would be expected from the grounded detector tube between steep potential walls similar to a particle in a box. This approximately reciprocal energy dependence clearly shows that the ion spends most of the time in the caps, and not in the tube.^73)^ The charge *z* is obtained from the amplitude analysis based on Fourier transformation. For example, pyruvate kinase (PK) 16-mer ions of (PK_4_)_4_ with 72 *e* (*m*/*z*=13.5 k) and 100 eV/charge has the fundamental frequency *f*=13.9 kHz.^72)^

**Figure 12:**
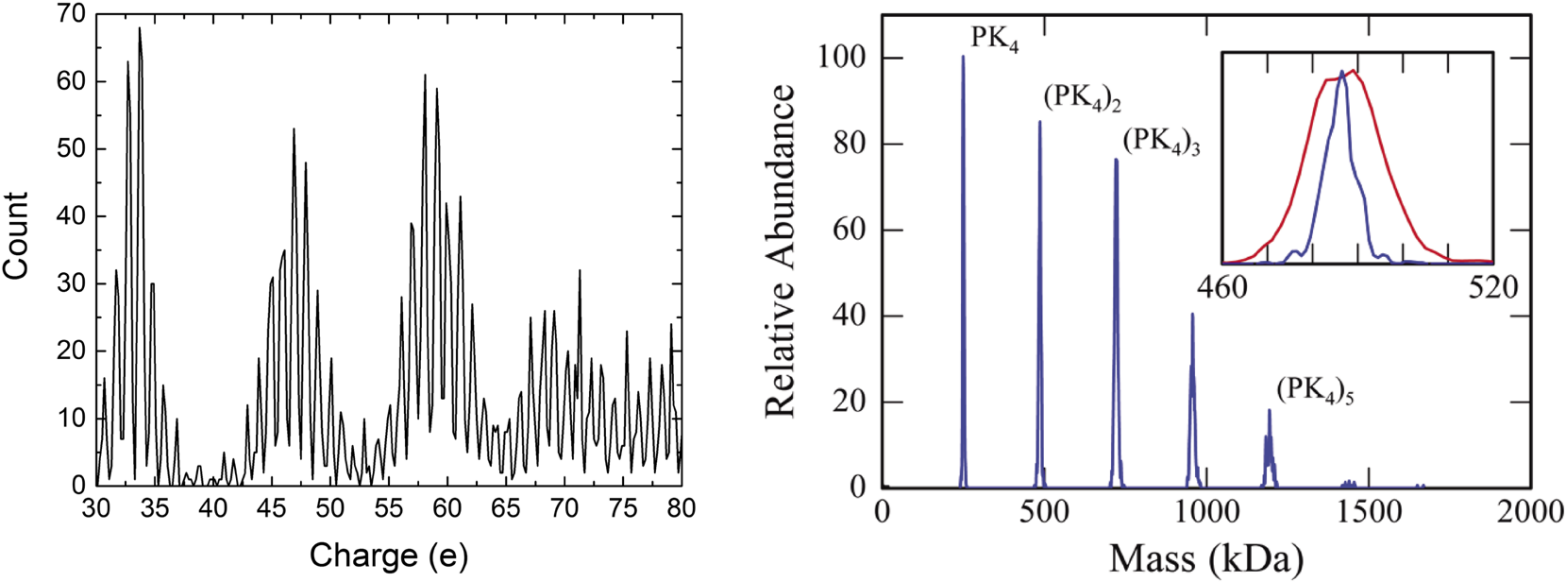
Fig. 12. (Left) Charge histogram for pyruvate kinase ions detected by charge detection mass spectrometry.^72)^ (Right) Mass histogram obtained with multiplying obtained *m*/*z* by *z*. The inset shows an expansion of octamer of (PK_4_)_2_ at 480 kDa.^72)^ Reprinted with permission from American Chemical Society. Copyright (2015) American Chemical Society.

Figure 12 (left) shows the obtained charge histogram of PK ions trapped for three seconds up to 60,000 oscillations where 3125 ions are collected.^72)^ The charge states are almost perfectly resolved with an uncertainty of less than 0.2 *e*. The superstructure with the peak at 33 *e* arises from its tetramer of PK_4_ . The other peaks centered at 47, 59, and 72 are due to (PK_4_)_2_*_,_*_3_*_,_*_4_, respectively. The mass histogram of the PK ions is constructed from the obtained information on the charge of *z* and the ratio of *m*/*z* by multiplying *m*/*z* by *z* (Fig. 12 (right)).^72)^ The inset of Fig. 12 (right) shows the expansion of (PK_4_)_2_ at 480 kDa. The blue and red lines show the mass peaks obtained with low (0.2 *e*) and high (0.65 *e*) charge uncertainty.^72,76)^ With strictly defined charge information, the mass spectra obtained by this method has a very high resolution even in high *m*/*z* ratios of around 10,000 with 485 kDa and +47 *e*. In the usual TOF, the obtained mass spectra appear not as separated peaks but as overlapped ones.^23)^ Much heavier samples such as viruses are also observed by this CDMS in which the mass and the charge reach the level of 25 MDa and 600 *e*.^34)^

To achieve a high sensitivity and accuracy especially in terms of charge, this measurement system requires conditions of a single ion trapped, no energy loss, and a long trap lifetime, some of which may be realized by a no collision condition.^72)^ In terms of single ion condition, the usual trap type mass spectrometer deduces *m*/*z* by Fourier transformation with multiple ions trapped which cause dephasing or correlation in ion motions. In this case the lifetime is short resulting in a low *S*/*N* and accuracy. Therefore, the proportionality between a signal amplitude and an intensity has been a serious problem.^68)^ To keep only single ion trapped in CDMS, the potential of the endcaps is manipulated periodically depending on whether ions are in or not.^71,75)^ The number of trapped ions is deduced by analyzing the detected signal. When the number is zero or more than one, the data is discarded. The probability of the number of the ions obeys a Poisson distribution, which strongly depends on the ion intensity. To maximize the probability for the single ion condition, the ion intensity is monitored and controlled.

It is difficult to realize a collision condition, even in an ultrahigh vacuum up to 3.6^−7^ Pa by a diffusion pump for preventing mechanical and electronical noise.^72)^ The mean free time is expected to around ms for a virus with a diameter of 16 nm under this pressure.^33,73)^ However the initial kinetic energy and mass are so large that collisions with light gas molecules such as nitrogen does not seem to disturb the oscillation in the trap. From the simple model of energy dissipation in thermal gas, the time constant for the energy loss would be predicted to be 3,000 s.^73)^ As the other path of the energy loss, the charging and discharging of the image charge (Fig. 9) have no contribution.^71,73)^ The energy loss in the one cycle is estimated to be Δ*E*=(*ze*)^2^/*C*, where *z* is the charge number of the ion in the trap and *C* is the summed capacitance connected to the tube detector. Applying *z* of 100 and *C* of 10 pF, we get the energy loss of Δ*E*=2.6×10^−23^ J=0.16 meV which can be neglected with the initial kinetic energy of 10 keV even in ten thousand cycles.

However some kinds of serious collisions clearly cause ion loss,^71)^ frequency shifts,^77)^ and charge loss.^73)^ The collisions are not serious in terms of the kinetic energy of the oscillating ions but they clearly limit the performance of CDMS. Investigations of the lifetime dependence on collisions revealed that multiple collisions such as three collisions for cytochrome *c* and four collisions for alcohol dehydrogenase are required to eject the ions from the trap.^71)^ The analyzed lifetimes and their molecular cross sections are in general agreement. The mechanisms for the requirement of multiple collisions are not fully understood but a trajectory shift in the trap caused by the collisions has been proposed for stability, which is described in the “Relationship between Charge and Mass or Size” sections.^77)^ The charge loss is also observed which may be caused by collisions.^73)^

Despite those difficulties, long and many cycle oscillations up to three seconds and 60,000 times have been realized.^72)^ With this multi-pass image charge detection, the charge evaluation of a single ion has been measured with almost perfect accuracy. The potential shift of the detector by the image charge is estimated to be 1 nV so that the *S*/*N* is not sufficient without an average.^73)^ Single-pass systems have also been reported.^28,74)^ However those systems tend to have a low sensitivity and low accuracy in terms of charge which is similar to the direct CDMS described above. Multi-detectors up to 22 are used to improve the *S*/*N* of the single-pass system^74)^ but the trap system shows much better performance. The noise is reduced as 
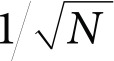
 where *N* is number of average or number of cycle in this system.^58)^ Actually charge uncertainty is expressed as Δ*z*=83.33*f* ^−0.458^ for 91 ms trap whereas *m*/*z* uncertainty is fixed as Δ(*m*/*z*)/*m*/*z*=0.008.^34)^ The *m*/*z* uncertainty is originated in mainly the energy spread of the energy selector and the trajectory spread in the trap.^71)^

With this sophisticated system, simultaneous measurements of mass and charge on very heavy ions can now be accomplished.

#### Other approaches

There are several approaches for simultaneously detecting charge and mass. Here two examples are introduced. The first one is kinetic energy detection by means of a superconducting detectors.^1,11,12,14,78)^ Ions accelerated under a potential difference have the kinetic energy proportional to their charge. With this method, it is possible to distinguish N^+^ and N_2_^2+^ . This method provides one of the most sensitive charge detectors. Actually, ions of multimers of polystyrene molecules with the mass up to MDa have been observed by this detector.^11)^ However, because of the low temperatures for superconductivity, it is very difficult to use them for general purposes. In terms of charge, the resolution is around 10. Energy resolution and linearity are not so high. However direct energy measurements could be expanded to detect their internal energy to distinguish whether the molecule is hot or cold.

The other one is based on fabricated mechanical device technologies.^79,80)^ Nanomechanical resonators with the size of several hundreds of nm detect and measure the mass of tantalum clusters.^79)^ The clusters with a mass of from 1.3 to 3.4 MDa and charge from 1 to 3 *e* are produced by a sputtering gas aggregation, which are monitored by a TOF and a transmission electron microscopy. The clusters are deposited on the resonator, resulting in a slight shift in the resonant frequency from several tens of MHz by tens of kHz. The shift is sufficiently large to detect and evaluate the mass of the clusters. In this study, the mass of the neutral clusters was determined to have almost the same mass as the charged ones. The high sensitivity for less than 500 kDa and the high resolution around 70 kDa are achieved. This method can detect neutral species which are still a challenging subject for MS. However the method can detect only a part of the clusters, about one particle out of a hundred million, due to the small cross section of the resonator. The sensor, in addition, may be used only at once because it would be difficult to remove the deposited particles from the sensor. These problems can be overcome by integration of many devices and parallel operation of them. The charge of around 1,000 *e* on a toner particle, on the other hand, is also measured by these kinds of devices: a cantilever of atomic force microscopy through the detection of Coulomb image force between the particle and the cantilever.^80)^

## RESULTS AND DISCUSSION

### Relationship between charge and mass or size

One of the fundamental challenges in MS is to quantitatively elucidate the relationship between mass and charge. For industrial interests, the charged particles are treated in terms of particle manipulation such as toner for electrophotographic applications, electrostatic deposition, and electrostatic precipitator for prevention of dust explosion and for the preparation of clean rooms.^81,82)^ For fundamental research in MS, charge evaporation and the Coulombic explosion of highly charged large ions have been studied to reveal ionization processes of ESI.^2,21,83)^ In the early stage of ion production processes of ESI, the size of the charged particles produced at the capillary shrink due to solvent evaporation. In the process, the charge is constant until the size reaches a limit: the Rayleigh limit^84)^ and the ion emission limit.^26,27,83,85)^ The number of charge corresponding to the Rayleigh limit (*n*_R_) and the ion emission limit (*n*_IE_) depend on the diameter of spherical droplets (*D*) as 
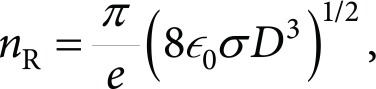
(4)
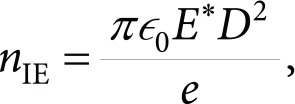
(5) where σ, E*, and *ϵ*_0_ are the effective surface tension of the droplet, the critical electric field on the droplet surface emitting ions, and vacuum permittivity, respectively. As a function on the diameter *D*, *n*_R_∝*D*^1.5^ and *n*_IE_∝*D*^2^. So in the larger size region *n*_R_ dominate the charge whereas *n*_IE_ in the smaller region.^24,27)^

Those limits have been investigated through simultaneous measurements of charge and size. In the larger size region of micro-droplets, ethylene glycol droplets produced by a piezoelectric injector with a radius of 50 μm in an ion trap are investigated *via* charge and size relationships by the light scattering for size evaluation and by levitation voltage scanning for charge and gravity evaluation.^26)^ During the trapping of several hundreds of seconds, particle size gradually shrinks but the charge is abruptly decreased by up to several tens of percent suggesting Coulomb explosion. The relatively large size make it possible to directly measure particle size by light scattering. When the particles are stabilized, the charge is much smaller than the limit.^35)^ A similar investigation was performed by direct and indirect CDMS on the charged polystyrene particles produced by ESI with various solution,^28,30)^ and by LIAD with corona discharge.^40,41)^ The maximum charge of polystyrene particles produced by ESI with water/methanol (50 : 50, v/v) depend on their mass within the Rayleigh limit 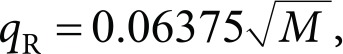
(6) where *q*_R_ is the number of charge corresponding to the Rayleigh limit, and *M* is the mass of the particle in Da.^30)^ Here, the density of the particle and surface tension of the solution are assumed.^28,30)^ The CDMS observation reveals that the limit dependence holds on the particles with diameters from 78 to 143 nm where the average charge is 70–80% of the limit.^30)^ On the other hand, the maximum charge is much smaller in the case of LIAD (Fig. 3). The maximum charge is around 2.0×10^5^ for a particle with a diameter of 15 μm and an estimated mass of 1.1×10^15^ Da, which would be 2.1×10^6^ in ESI from Eq. (6). In spite of the less maximum charge, the diameter dependence from 3 to 29.6 μm of polystyrene particles with LIAD shows that the average charge varies from 4,250 to 1.3×10^4^ following the relationship between charge *q*_AV_ and diameter *D* as 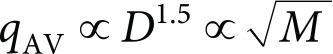, which is the same as the dependence of ESI (Eqs. (4) and (6)). The agreement suggests that similar processes restrict the maximum charge on particles in ESI and LIAD with corona discharge.

To investigate much smaller region, globular proteins are studied by ESI-MS assuming native structures.^83)^ The obtained charge states of the proteins are much smaller than those expected from the Rayleigh limit of Eq. (4) and the dependence of charge on the diameter of a protein is in good agreement with the ion emission model of Eq. (5) suggesting the existence of the ion emission processes. From the data fitting to Eq. (5), a few critical electric fields of E* and their dependence on the size are obtained, which also supports the ion emission processes. In spite of these successful results, the study again shows the limit of MS: assumed structures and restricted size controllability. We cannot use predefined large particles with a large charge because we cannot identify the charge and the mass simultaneously only from *m*/*z*. The samples, then, are restricted to the smaller globular proteins with well know structures.

**Figure 13:**
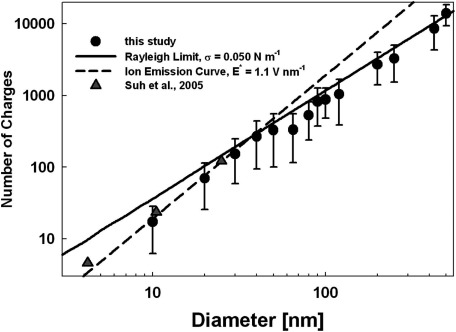
Fig. 13. Relationship between charge and size of predefined particles of polystyrene, polyethylene glycol, sucrose, and metals with the diameter from 10 to 500 nm, which are ionized by ESI with their suspension in 50% methanol, 50% water, and 1 mM ammonium acetate.^27)^ Reprinted with permission from American Chemical Society. Copyright (2009) American Chemical Society.

The limit can be overcome with IMS measurements performed using the tandem system with neutralizer described in the “Ion Mobility Spectrometry” section.^24,27)^ Predefined polystyrene, polyethylene glycol, sucrose, and metal particles with a diameter from 10 to 500 nm are ionized by ESI when suspended in water/methanol (50 : 50, v/v) and 1 mM ammonium acetate followed by a simultaneous charge and size measurement with the tandem IMS. Figure 13 shows the thus obtained plot of charge *versus* size. Clearly the size dependence changes at around 40 nm suggesting a change in criteria between the Rayleigh limit and the ion emission limit. In the larger region more than 40 nm, the charge dependence fits well to Eq. (4) and consequently to Eq. (6) with conversion from *D* to *M* .

Those results are consistent with the ESI processes alluded to above. However, the discussion on the obtained relationship between charge and size assumes perfectly spherical droplets which may not be realistic.^2)^ With structural deformation of the droplets, which could be caused by structural vibrations, collisions, or external electric fields, acute or high-field positions like a vertex of a Taylor cone should be produced where the asymmetrical fission or ion emission is preferred.^2)^ However it is very difficult to discern the details of the ESI processes, especially for rapid charge loss processes, because light scattering and IMS are “slow” measurements revealing only the stability of the final products. Measurements on mass *versus* charge have been not been performed on the emitted ions with small mass and charge but, rather, on the final ones with large masses and charges. High-speed observations both on the emitted ions and the final products have been difficult.^2,73)^

The indirect CDMS with the ion trap is suitable method on this subject. With an oscillation frequency of tens of kHz, the sensitivity of single ion detection, and the high resolution both on mass and charge, small ion emissions from virus ions with the charge of 146 *e* and the mass of 4 MDa are observed.^73)^ The emission is actually observed through the change of the oscillation frequency of 10 kHz with the shift around 50 Hz because the amplitude corresponding to the charge is less reliable due to the noise. Careful analyses have been performed on the processes involving collision with buffer gas, solvent evaporation, ion emission, and Coulomb interaction between ion and image charge. The frequency shift is caused by one charge emission with mass of 1,000 Da. Total mass loss is 20 kDa due to solvent evaporation in three seconds. Most of ions (43%) have at least one emission while a few of them (11%) have twice or more emissions in three seconds. The emission is promoted by the electric field in the trap. CDMS cannot only characterize mass and charge of the heavy and highly charge ions but also track the change of them with high time resolution.

### Structural analyses of large system

**Figure 14:**
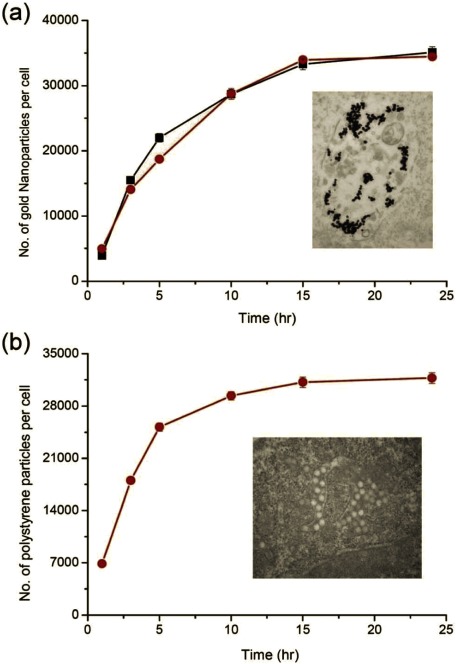
Fig. 14. Cellular uptake of (a) 50 nm gold particles by NTERA2 cells and (b) 100 nm polystyrene particles by Raw264.7 cells.^4)^ As the incubation time increases, the number of the particles uptaken by the cells increase, which are observed by direct CDMS (red circles) and by ICP-MS (black squares). The measurements by ICP-MS are performed not for polystyrene particles but for gold particles. The insets show TEM images of the cells with the particles.^4)^ Reprinted with permission from Royal Society of Chemistry. Copyright (2013) Royal Society of Chemistry.

With the methods mentioned above, large systems such as cells, viruses and nano composites are within the scope of MS. The cellular uptake of nano/microparticles has been investigated by the direct CDMS with LIAD.^4)^ NTERA2 cells are incubated with gold nanoparticles with diameters of 30 and 250 nm. The observed mass of the cells increases with increasing incubation time. The mean mass increases 60% and 200% with the 30 nm and the 250 nm particles, respectively.^4)^ Figure 14 shows the incubation time dependence of the average numbers of uptaken (a) 50 nm gold particles by NTERA2 cells and (b) 100 nm polystyrene particles by Raw264.7 cells and the corresponding images of transmission electron microscopy (TEM).^4)^ The red circles and lines of Figs. 14a and 14b represent the particle numbers observed by direct CDMS while the black squares and lines of Fig. 14a represent those observed by ICP-MS. Both results are in good agreement with each other showing that direct CDMS is as reliable as ICP-MS in terms of the quantitative measurements. TEM measurements also supports the cellular uptake of the gold particles. However ICP-MS cannot be applied to polystyrene particles because the method is strongly dependent on key elements such as gold. In this case, TEM is used as a unique measurement method, which has less reliability as the quantitative measurements. CDMS, in turn, can detect the uptake of polystyrene particles by directly observing the mass of cells (Fig. 14b). Here the features of MS, such as material independence and quantitative measurements, are still valid in CDMS.

**Figure 15:**
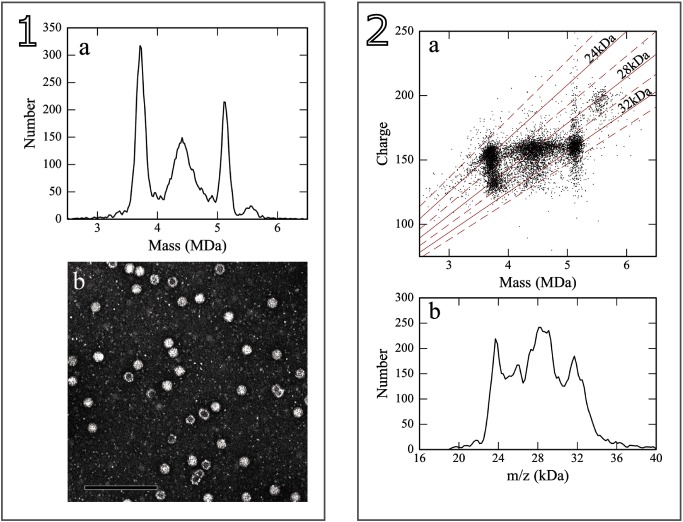
Fig. 15. 1. Mass spectrum and TEM image of recombinant adeno-associated viruses (AAV) capsids with scDNA genomes.^86)^ The three peaks in the mass spectrum corresponds to empty, partially filled and fully filled with scDNA. The TEM image shows uniform white filled spots and white rings as filled and empty ones (scale bar=200 nm). 2a. Charge *versus* mass scatter plot.^86)^ 2b. *m*/*z* histogram.^86)^ Reprinted with permission from American Chemical Society. Copyright (2016) American Chemical Society.

Figure 15 shows mass spectra and a TEM image of virus capsids encapsulating scDNA.^86)^ A capsid is the protein shell of a virus assembled from the capsid viral protein (VP). The sample used here is recombinant adeno-associated viruses of serotype 8 (AAV8), which are promising vectors for human gene therapy. AAV is constructed from 60 copies of three different VPs (VP1, VP2, and VP3). VP1 is the longest with 738 residues and mass of 81,624 Da while VP2 and VP3 are shorter with mass of 66,649 and 59,762 Da. The mass spectrum (Fig. 15-1a) shows three peaks at 3.704±0.013, 4.4, and 5.095±0.025 MDa. The lightest one is empty AAV8, which is in good agreement with the expected mass of 3.729 MDa from the already known stoichiometry of V1 : V2 : V3 of around 1 : 1 : 10. The heaviest one is filled AAV8 with scDNA. The mass difference between the filled and the empty ones is 1.392±0.013 MDa showing the expected sequence mass of the scDNA (1.389 MDa). The close agreement indicates that the scDNA is mainly packaged without counterions. The broad peak around 4.4 MDa is due to partially filled ones. The intensity ratio of each peak (empty:partial:filled) is 35 : 42 : 23 which is consistent with that obtained from the TEM image where uniform white filled spots and rings represent filled and empty ones, respectively (Fig. 15-1b).

The charge *versus* mass scatter plot of the same sample clearly show that there is large charge distribution (Fig. 15-2a).^86)^ Two charge states are found in the empty capsids, which are due to differences in compaction.^87)^ The compact capsids without defects have a perfect icosahedral structure and have less chance to acquire a charge in ESI processes.^87)^ The empty capsids with some defects have a slightly larger charge. The filled ones, on the other hand, have much wider charge distribution than that of empty ones suggesting not compact structures because of intervene of the encapsulated scDNA.

Similar structural studies on devastating pathogen viruses such as hepatitis B virus (HBV) have been performed. The building block for HBV capsid is core protein assembly domain Cp149 dimer. The capsid consists of 90 or 120 dimers with icosahedral structures. The icosahedral structures are observed by TEM with spherical shapes, but it is difficult to evaluate intermediate and structures with defects. With CDMS, the structures of intermediate are investigated. From the perfect icosahedral structure with 120 Cp149 dimers, 3 to 16 dimers are lost, thus creating a hole on the shell, which are also observed by TEM.^33)^

Figure 15-2b shows an *m*/*z* spectrum constructed from the charge *vs.* mass scatter plot by scanning diagonal lines in Fig. 15-2a.^86)^ Because the averaged charge is almost same, some information on three different species with different masses can be obtained as three major peaks in the *m*/*z* spectrum of Fig. 15-2b. However there are many minor peaks arising from widely distributed charge states and the relative abundances are totally different from those obtained from the mass spectrum. CDMS will play crucial roles in the quantitative abundance evaluation and defect evaluation, which are generally difficult for other methods.

**Figure 16:**
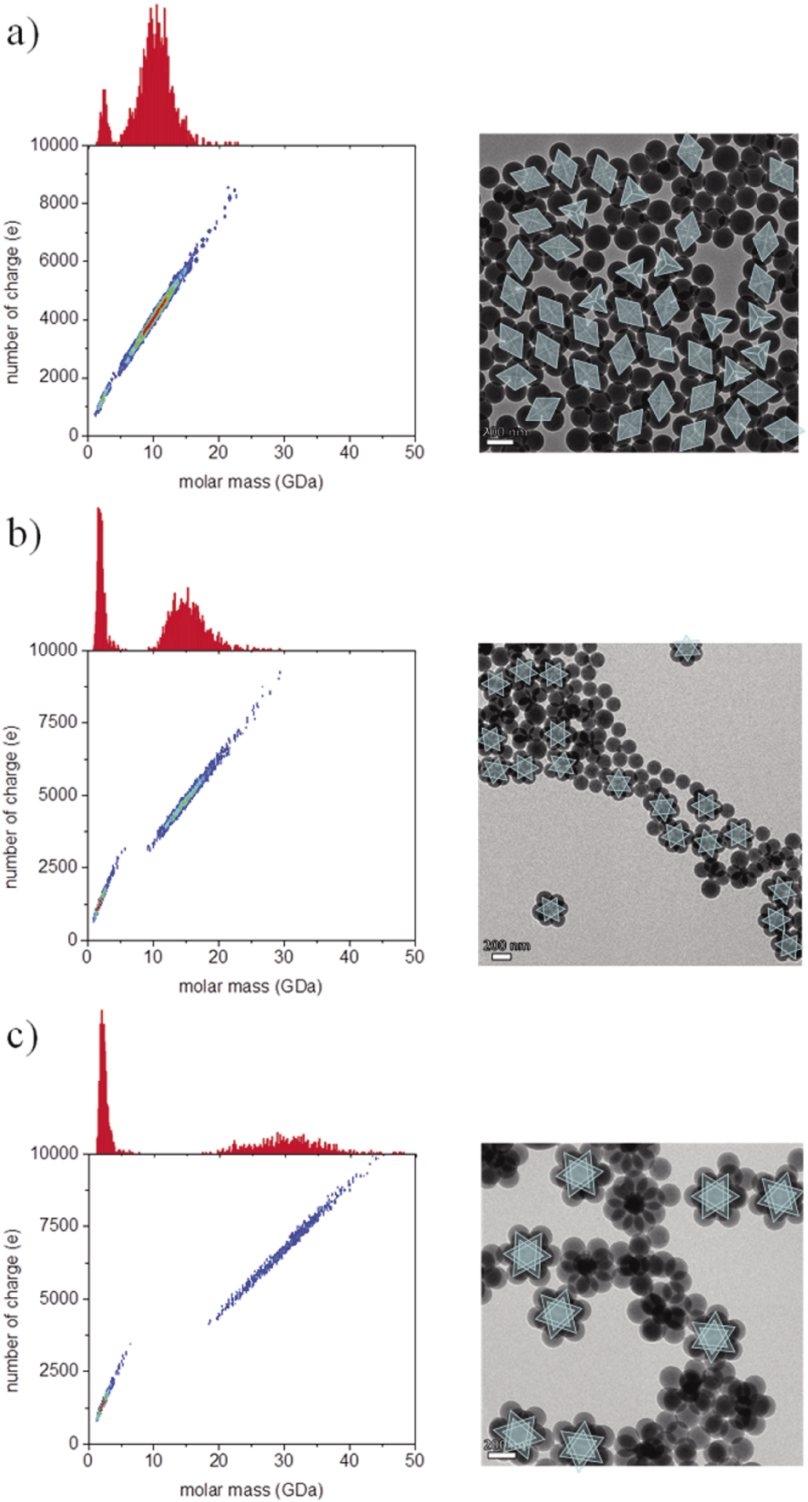
Fig. 16. Relationship between charge and mass of polystyrene/silica multipods and corresponding TEM images.^31)^ The scale bar is 200 nm. Reprinted with permission from American Chemical Society. Copyright (2015) American Chemical Society.

Figure 16 shows CDMS spectra of polystyrene(PS)/silica multipods and corresponding TEM images.^31)^ The particles have composite structures with center silica particles surrounded by PS particles. The composite structures of tetrapods (TP), hexapods (HP), and dodecapods (DDP) are mainly controlled by the size of center silica particles, which are shown in Figs. 16a, 16b, and 16c, respectively. Apparently each charge *vs.* mass scatter plot of Fig. 16 shows two components, corresponding to the free PS particles and the composite particles. The mass of each component is 2, 10.4, 15.5, and 30.4 GDa, for free PS, TP, HP, and DDP, respectively. The relative proportions obtained by TEM and CDMS are in good agreement with each other. To elucidate relative proportion, mass information is more reliable than that of TEM because the number of detected ions are much larger than that of analyzed images, and because images are sometime difficult to be analyzed. It is possible to quantitatively analyze much larger numbers of samples with CDMS, which cannot be treated by the usual MS. Furthermore, the slopes in the spectra, charge *versus* mass, decrease from TP (Fig. 16a) to DDP (Fig. 16c) suggesting “accessible surface area” (ASA) of the composite nanoparticles. Since DDP has a compact structure compared to that of TP, the fact that charge/mass of DDP is smaller than that of TP can be explained by the difference in ASA. The detailed structure of those relatively large system can be studied by CDMS.

Not only mass and charge but also cross sectional information can be obtained by CDMS, which can be used to directly explore structures of heavy molecules.^77)^ Cytochrome *c*, ubiquitin, and Bovine serum albumin (BSA) ions are produced by ESI with charge of +15, +10, and +50 *e*, respectively. They are introduced in CDMS with the energy of 202.4 eV/charge at a pressure of 5×10^−9^ Torr. In the trap of CDMS, ions collide with background gas, which leads to frequency shifts. From the amount and the intervals of the frequency shifts, the collision cross section can be evaluated. This trap type CDMS can track the changes of charge, mass, energy, and the cross section for long-term up to several seconds with high time resolution up to tens of μs. This feature should be emphasized. As described in the “Relationship between Charge and Mass or Size” sections, the frequency of the ion in indirect CDMS is sensitive to almost every characteristic of the ion, including energy, mass, charge and so on.^73)^ In addition, the frequency dependence on those characteristics is determined by the design of the trap. For example, the frequency dependence in this study on the ion kinetic energy (*E*) is represented as^77)^

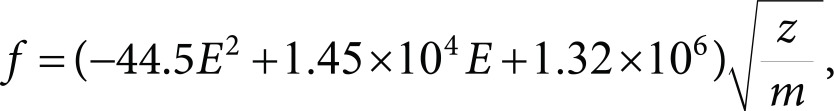
(7) which is totally different from that of Prof. Jarrold’s group represented by Eq. (3).^73)^ Careful analyses must be performed to elucidate the nature of the frequency shift and to deduce the information of ions.^73,77)^

## CONCLUSION

Recent topics on mass and charge measurements on heavy ions are introduced in terms of technical issues and ongoing results. The concepts and basic techniques have been developed for long time but recent progress makes it possible to simultaneously detect the mass and charge of single heavy ions. With this method, large systems such as cells and nano composites are within the scope of MS. Quantitative, statistical, and material independent evaluations will widely be used in various field. However the need for many improvements is inevitable and the most urgent one is throughput.

As described above charge measurements must be performed on every single ions in CDMS regardless of the way of direct or indirect. To deduce statistically meaningful information, as many as ions, say, up to ten thousands should be observed. The situation is the same even in the case of conventional MS but the precise charge detection requires long measurement time resulting in low throughput. Low throughput is particularly serious in indirect CDMS. Because a single ion must be trapped for long time up to several seconds to achieve high charge sensitivity and accuracy, the throughput for the whole measurement is low with substantial time being required to permit information on many ions, *i.e.*, up to ten thousand, to be accumulated. The number of ions and the frequency used in CDMS are one and tens of kHz, respectively, which are much smaller than those of FTICR or Orbitrap™.

Even with those difficulties, there are many ways to improve performance. Conventional MS widely contributes to various fields with high sensitivity, throughput, and resolution but within the low mass region. CDMS is opening a new field that cannot be accessed by conventional MS. There is no doubt that future devices will be equipped with commercial mass spectrometers and will be used to collect further information on complicated systems such as living organisms and real catalysts.

## Notes

Mass Spectrom (Tokyo) 2017; 6(3): S0074
